# Responses of Seed Germination, Seedling Growth, and Seed Yield Traits to Seed Pretreatment in Maize (*Zea mays* L.)

**DOI:** 10.1155/2014/834630

**Published:** 2014-06-26

**Authors:** Yu Tian, Bo Guan, Daowei Zhou, Junbao Yu, Guangdi Li, Yujie Lou

**Affiliations:** ^1^College of Animal Science and Technology, Jilin Agricultural University, Changchun, Jilin 130118, China; ^2^Key Laboratory of Coastal Environmental Processes and Ecological Remediation, Yantai Institute of Coastal Zone Research, Chinese Academy of Sciences, Yantai, Shandong 264003, China; ^3^Northeast Institute of Geography and Agricultural Ecology, Chinese Academy of Sciences, Changchun, Jilin 130102, China; ^4^Graham Centre for Agricultural Innovation (Alliance between NSW Department of Primary Industries and Charles Sturt University), Wagga Wagga Agricultural Institute, Pine Gully Road, Wagga Wagga, NSW 2650, Australia

## Abstract

A series of seed priming experiments were conducted to test the effects of different pretreatment methods to seed germination, seedling growth, and seed yield traits in maize (*Zea mays* L.). Results indicated that the seeds primed by gibberellins (GA), NaCl, and polyethylene glycol (PEG) reagents showed a higher imbibitions rate compared to those primed with water. The final germination percentage and germination rate varied with different reagents significantly (*P* < 0.05). The recommended prime reagents were GA at 10 mg/L, NaCl at 50 mM, and PEG at 15% on account of germination experiment. 15% PEG priming reagent increased shoot and root biomass of maize seedling. The shoot biomass of seedlings after presoaking the seeds with NaCl reagent was significantly higher than the seedlings without priming treatment. No significant differences of plant height, leaf number, and hundred-grain weight were observed between control group and priming treatments. Presoaking with water, NaCl (50 mM), or PEG (15%) significantly increased the hundred-grain weight of maize. Therefore, seed pretreatment is proved to be an effective technique to improve the germination performance, seedling growth, and seed yield of maize. However, when compared with the two methods, if immediate sowing is possible, presoaking is recommended to harvest better benefits compared to priming method.

## 1. Introduction

In semiarid area, seasonal drought is often frequent in spring and autumn, especially in the sowing season. Soil evaporation will lead to a large amount of moisture loss, of which 90%–95% occurred in 5–10 cm soil layer [1], that is, the optimum depth for crop sowing. Under this condition, it is important to improve the water use efficiency of crop seedlings or find some ways to increase crop yield under drought conditions [2].

Maize (*Zea mays* L.) is an important crop in the world; it is widely used for feed and industrial raw material. Maize ranks the third in world production following wheat and rice for the area and production. It is also the main crop in northern China, where the climate is a combination of temperate and semiarid monsoon. Rapid and uniform field emergence is an important factor to achieve high yield to meet the growing demand for food [3].

Seed priming is a presowing treatment that exposes seeds to a certain solution that allows partial hydration but not germination [4], and redried to original moisture content. Although the germination is not completed, metabolic activities that prepare seeds for radicle protrusion may be initiated during priming [4, 5]. Many evidences have shown seed priming could improve germination and early seedling growth under stress conditions compared to plants grown from untreated seed [6–8].

Various priming treatments have been developed to increase the speed and synchrony of seed germination [6, 9, 10]. Common priming techniques include hydropriming (soaking seed in water), osmopriming (soaking seed in osmotic solutions such as PEG), halopriming (soaking seed in salt solutions), and priming with plant growth hormones. However, different priming effects were reported with different priming reagents and species. For instance, when* Lolium perenne* seeds were primed with PEG solution, the germination was significantly improved, but no obvious effects were observed with* Festuca rubra*,* Festuca ovina,* and* Poa trivialis* [11]. Seed priming with optimal concentrations of plant growth hormones, such as auxin (IAA), gibberellins (GA), abscisic acid, and ethylene, has proven that germination performance as well as growth and yield of many crop species under both normal and stress conditions could be improved effectively [12, 13]. By soaking seeds (sorghum, rice, or wheat) in water and planting the same day (so-called presoaking treatment), the germination rate could also be increased and seedling emergence improved [14].

In recent years, numerous studies were devoted to the physiological responses of seed germination and seedlings stages to chilling or osmotic stress [8, 13]; the ecological responses of the whole growing season remain largely unknown. To elucidate the ecological responses of different pretreatment to maize species, it may be useful to investigate the changes in not only germination stage, but also seedling growth and yield responses. Few studies to date have attempted to test the whole growing season response to different pretreatments. In this study, we choose water, PEG, NaCl, and GA as different priming reagents, to investigate the dynamics of seed water uptake during seed priming, germination and seedling growth responses after seed priming and presoaking, and yield response to different pretreatment.

## 2. Materials and Methods

Four experiments were conducted at Jilin Agricultural University. Seeds of maize (*Zea mays* L.) cv. Jinong 610 were used as test materials. All seeds were pretreated with 0.1% H_2_O_2_ solution for 5 min and then thoroughly washed for 5 min prior to seed treatments.

### 2.1. Experiment 1: Seed Priming Experiment

There were 10 treatments with 4 seed pretreatment reagents, namely, water, NaCl (50, 150, 250 mmol/L), PEG (10%, 15%, 20%), and Gibberellin (GA) (5, 10, 15 mg/L), and unprimed seeds were used as control, replicated 4 times.

Thirty seeds were placed in two layers of filter paper in a 12 cm Petri dish. The filter paper was moistened with about 30 mL of different priming reagents, ensuring that the seeds were immerged with solutions. Seeds were primed in different priming reagent solution in the lab with room temperature 14°C to 21°C and relative humidity 48% to 64% at night and during day, respectively.

At the hydration stage, the seeds were weighted every 4 h after the surface solutions were dried with filter paper until the weight of seeds was not changed (seeds were saturated). On the dehydration stage, seeds were placed in dry Petri dishes and weighted every 4 h to original weight [15].

The water content of seeds and rate of hydration was calculated with the following formula:

(1) Water  content=W1−W0,Imbibition  rate=(W1−W0)W0,

where *W*_1_ is the weight when the seed was saturated with different solutions and *W*_0_ is the original weight.

### 2.2. Experiment 2: Germination Experiment

The germination experiment was conducted at growth chambers (HPG-400, Haerbin, China) at relative humidity of 60% with 12 h photoperiod (Sylvania cool white fluorescent lamps, 200 mmol m^−2^ s^−1^, 400–700 nm, 25/15°C).

The primed seeds under different priming reagents from Experiment 1 were germinated in Petri dishes (12 cm diameter) containing two layers of filter paper with 15 mL of distilled water. Each Petri dish contained 30 seeds representing an experimental unit. The seeds were considered to have germinated after radicle emergence. Germination test was ended when no seeds have germinated for 3 days. The germination period was 12 days.

The rate of germination was estimated using a modified Timson's index of germination velocity = Σ*G*/*t*, where *G* is the percentage of seed germination at one-day intervals and *t* is the total germination period [16]. The maximum value possible for our data using this index was 100 (i.e., 1000/10). The greater the value, the more rapid the rate of germination.

### 2.3. Experiment 3: Seedling Response to Seed Treatments

There were two seed treatment methods (priming and presoaking) and 4 seed treatment regents (water, 50 mM NaCl, 15% PEG, or 10 mg/L GA) with untreated seeds as control. The experiment was a 2 × 4 factorial design, replicated 5 times.

Seeds were either primed as described in Experiment 1 or presoaked using regents mentioned above. Those solutions were selected based on the results from Experiments 1 and 2, which were the best expressive concentrations of each reagent to prime the seeds.

The experiment was conducted in a glasshouse but partially shaded under maximum photosynthetically active radiation of 1000 *μ*mol m^−2^ s^−1^, day/night temperature of 30/24 ± 3°C. Seeds were sown in 25 cm diameter plastic pots that contained 4 kg of native loamy soils. Ten seeds were sown with 3 cm depth in each pot and then thin to 5 seedlings after geminating. Pots were destructively harvested 40 days after seed germinated.

Seedling height, root length, and the biomass of different organ parts were measured at harvest.

### 2.4. Experiment 4: Yield Response to Seed Treatments

The experiment was conducted in the field. Seeds were pretreated as mentioned in Experiment 3. The experimental design was identical to Experiment 3, but replicated 3 times.

Two or three seeds were sown every 40 cm at the bottom of the ridge and then covered with about 5 cm of soil in a 50 m long row as one plot. At the three-leaf stage plants were thinned to one per hole. Each plant received 5.3 g urea/per plant (200 kg urea/ha) on the surface of the soil 10 cm from the plant when it reached the eighth leaf stage. Weeds, insects, and diseases were controlled adequately when necessary.

Ten successive plants per plot were randomly selected at physiological maturity stage. The leaf number and shoot height were measured before harvest. Grain yield and 100-grain weight were determined by oven drying samples at 65°C. Yield was expressed as t/ha.

### 2.5. Data Analysis

Analysis of variance (ANOVA) was conducted using Statistics SPSS 19.0. Water intake and imbibition rate in Experiment 1 were analyzed using repeated measures. One-way ANOVA was performed for data from Experiment 2 and two-way ANOVA was employed for data from Experiments 3 and 4. The treatment mean values were compared with the least significant difference (LSD) at the 5% level.

## 3. Results

### 3.1. Experiment 1: Seed Water Intake under Priming Cycle

**Figure 1:**
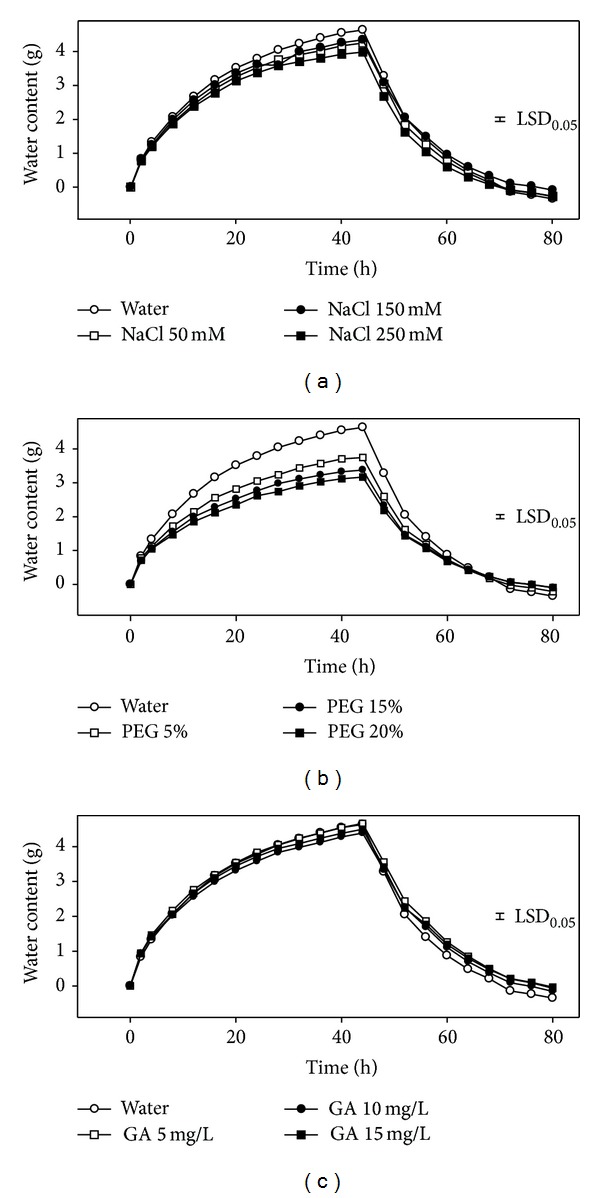
Effects of pretreatment reagents on water intake under priming cycle (*n* = 4).

The water intake of maize seed showed similar trends in different priming reagents. The percentage of water intake was greater at the first 12 hours then slowed down. It took about 44 h for the seeds to be saturated (Figure 1). When comparing different concentrations of different solutions with water (control), the water intake with 250 mM NaCl was significantly lower than control, and all the three concentrations of PEG showed slower water intake than control. No significant differences were observed between GA and control. One hydration-dehydration cycle lasted about 84 h.

**Figure 2:**
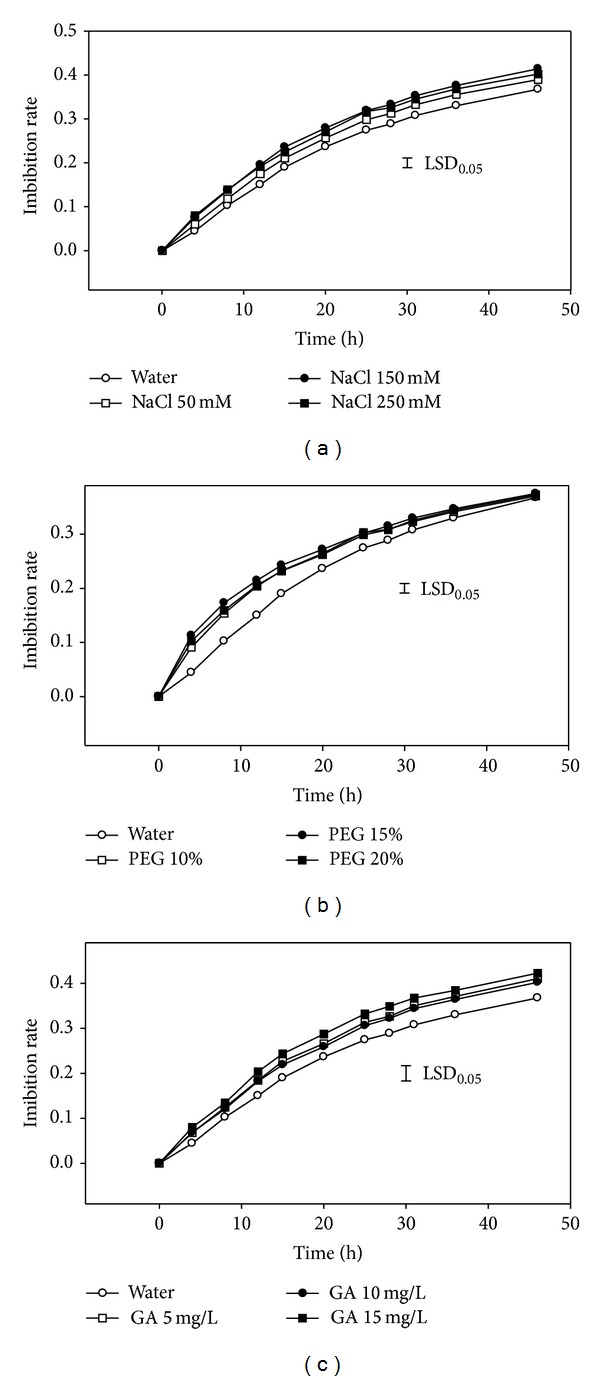
Effects of pretreatment reagents on imbibition rate after seed priming (*n* = 4).

The imbibitions rate of seeds was increased after one hydration-dehydration cycle (Figure 2). Compared with water priming, the seeds showed a higher imbibitions rate when primed by GA, NaCl, and PEG reagents. The imbibitions rates were significantly higher when primed with NaCl (150 and 250 mM), GA (15 mM), and all the three concentrations of PEG compared with those primed in water (Figure 2).

### 3.2. Experiment 2: Germination Responses after Seed Pretreatment

**Figure 3:**
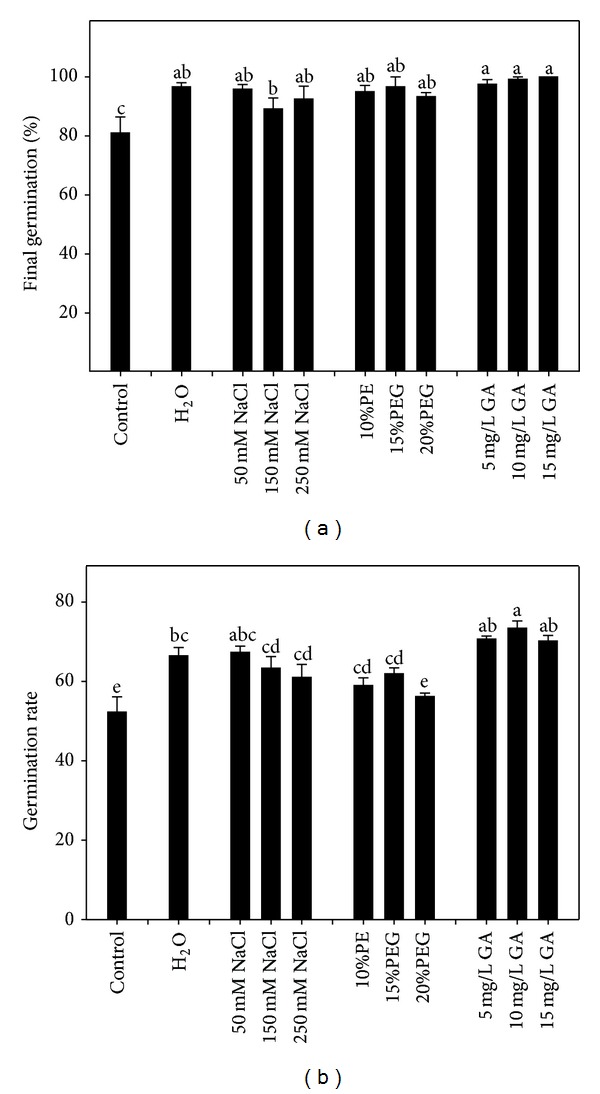
Effects of pretreatment reagents on final germination percentage and germination rate of maize (*n* = 4).

The seed pretreatment of different reagents had significant (*P* < 0.05) effects on the final germination percentage and germination rate (Figures 3(a) and 3(b)). Primed seeds had significantly higher germination percentage than those in control, but no significant differences were observed among priming reagents. The germination rate was also significantly increased by seed priming except 20% PEG. GA showed greater effect on germination rate, and PEG had slightly increased germination rate when compared with other reagents. No statistical differences were observed between water and NaCl priming methods (*P* > 0.05).

Within each reagent, the concentration of different priming reagents also showed different effects to germination percentage and germination rate. The optimum germination performance was observed after priming with 10 mg/L GA, 50 mM NaCl, and 15% PEG, which were used for Experiments 3 and 4.

### 3.3. Experiment 3: Seedling Response to Seed Pretreatments

**Figure 4:**
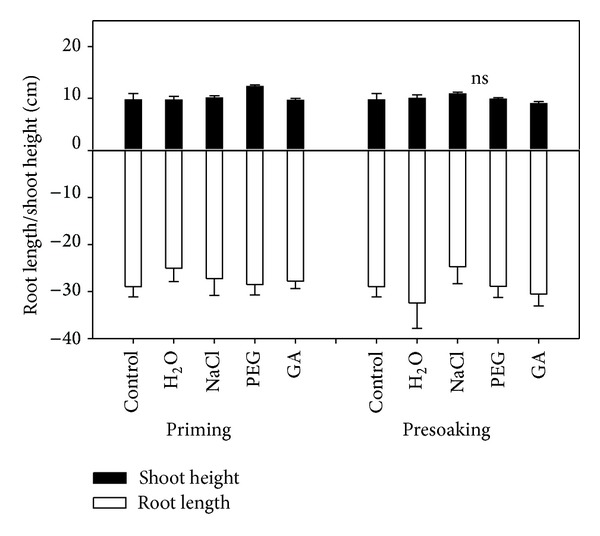
Effects of pretreatment reagents and priming methods on seedling shoot height and root length of maize (*n* = 10; ns: not significant).

**Figure 5:**
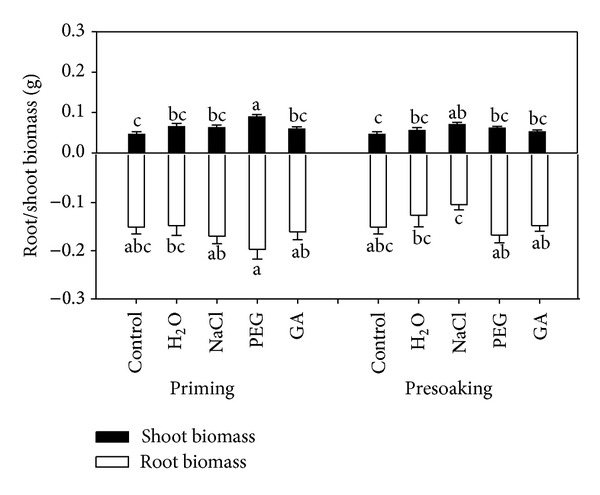
Effects of pretreatment reagents and priming methods on seedling shoot and root biomass of maize (*n* = 10).

No significant differences were observed in plant height and root length after priming by different reagents (Figure 4). The shoot and root biomass of maize seedlings were significantly affected by priming reagents (Figure 5 and Table 1). Pretreatment methods (priming and presoaking) also significantly affected the root biomass (*P* < 0.05). The shoot and root length showed no remarkable differences by the interaction of priming reagents and pretreatment methods in this pot experiment. Priming treatment with PEG reagent significantly increased shoot biomass compared to the control group. The shoot biomass was also significantly higher when presoaking with NaCl reagent compared to control. However, no significant differences were observed in root biomass between treatments.

### 3.4. Yield Response to Seed Pretreatment

There was no significant difference in plant height between seed pretreatments. Presoaking with PEG and GA significantly increased the leaf number when compared with control. No significant differences were observed to the seed yield and hundred grain weight between different priming reagents (Table 1). But compared with priming methods, presoaking with water, NaCl, or PEG significantly increased the hundred-grain weight of maize (Table 2).

## 4. Discussion

The study revealed that seed priming and presoaking techniques using different solutions can significantly improve maize plant performance by increasing seed germination rate, seedling biomass, and seed yield, although response varied with different solutions and concentrations. Seed priming with different concentrations of solutions significantly improved germination performance, but no remarkable differences were observed for seedling growth and seed yield, while seed presoaking with some solution dramatically improved seedling growth and seed yield of maize.

Germination and seedling establishment are critical stages which affected both quality and quantity of crop yields [17]. Soil water content is the key factor affecting seed germination and plant establishment in the semiarid area. The present study showed that, compared with the control group, the rate of hydration increased dramatically after seed primed by reagents. This implies that seed priming may improve seed germination of maize seeds by speeding up imbibition, which could contribute to facilitate emergence phage of maize after raining in the semiarid area. Similar results were also reported that priming improved germination of sunflower cultivars by accelerating imbibitions [18].

Generally, seed germination entails three distinct phases: (i) imbibition, (ii) lag phase, and (iii) radicle growth and emergence [19]. The purpose of priming is to prolong the lag phase, which allows some pregerminative physiological and biochemical processes to take place but prevents germination [20]. The results of our germination tests indicated that seed priming significantly increased the final germination percentage and germination rate of maize. The increment in seed germination due to seed priming treatment is in conformity with other researchers [21, 22]. When compared with different priming reagents, GA of all concentrations showed greater influence on germination rate. It was reported earlier that GA participated in regulation of many growth and developmental processes in plants [23, 24] and was particularly important in regulating stem elongation [25]. GA treated seed was closely associated with their rapid utilization in the synthesis of various amino acids and amides [26], which could be the reason for the increased germination rate.

Priming reagents (especially for PEG priming) had the beneficial effects on shoot and root biomass. This was mainly due to the accelerated metabolism occurring in primed seeds, which increases the imbibition speed as compared to unprimed seeds. Similar results were also reported by Guan et al. [27] on sorghum seeds. Farooq et al. [28] reported that presoaking with inorganic salts improved seedling emergence, shoot and root length, and biomass. In contrast, no significant differences were observed for plant height, root length, and biomass in the present study. Only presoaking with NaCl solution increased shoot biomass, which had a similar trend with the study by Farooq et al. [28].

Increasing evidence suggests that seed priming could change the crop performance from physiological, biochemical, and molecular aspects [8, 29]. There are several studies arguing that the priming itself could be a stress, for the water uptake activates previously quiescent cellular events in primed seeds while compromising the desiccation tolerance [30, 31]. Chen and Arora [31] also concluded that primed seeds, no matter what the priming scheme that was chosen, would inevitably endure some injury by the dehydration treatment. Only few studies reported that seed priming could increase the grain yield as shown by Sharifi and Khavazi [6] with plant growth promoting Rhizobacteria (PGPR). However, in the current study, no significant difference in grain yield was observed in priming treatments while presoaking with water, NaCl, and PEG solutions did significantly increase the grain yield. Harris et al. [32, 33] also reported that presoaking followed by surface drying has more advantages to yield in many field crops.

## 5. Conclusions

The results indicated that the final germination percentage and germination rate varied with different reagents significantly (*P* < 0.05). The GA at 10 mg/L, NaCl at 50 mM, and PEG at 15% were recommended on account of germination experiment. 15% PEG priming reagent increased shoot and root biomass of maize seedling. The shoot biomass of seedlings after presoaking the seeds with NaCl reagent was significantly higher than the seedlings without priming treatment. No significant differences of plant height, leaf number, and hundred-grain weight were observed between control group and priming treatments, while presoaking with water, NaCl, and PEG solutions did significantly increase the hundred-grain weight of maize. Our results confirmed that seed pretreatment is an effective technique to improve the germination percentage, germination rate, seedling growth, and seed yield. However, if immediate sowing is possible, presoaking is recommended to harvest better benefits compared to hydration–dehydration method.

## Figures and Tables

**Table 1:** Analysis of variance for seed priming effects on maize for seedling growth and yield composition (*n* = 10, *P* < 0.05).

Source	Seedling growth (pot experiment)				
	df	Shoot height	Root length	Shoot biomass	Root biomass
Priming reagents	4	0.152	0.588	0.000∗	0.012∗
Treatment methods	1	0.366	0.366	0.074	0.005∗
PR × TM	4	0.206	0.206	0.074	0.394

	Yield composition (field experiment)				
	df	Seed yield per plant		Hundred-grain weight	

Priming reagents	4	0.569		0.071	
Treatment methods	1	0.180		0.001∗	
PR × TM	4	0.673		0.047∗	

*P < 0.05.

**Table 2:** Effects of pretreatment reagents and priming methods on yield composition of maize (*n* = 10).

Pretreatment methods	Reagents	Plant height (cm)	Leaf number	Yield (*t*/hr)	Hundred-grain weight (g)
	Control	185.11 ± 3.36^a^	9.00 ± 0.49^bcd^	13.24 ± 1.57	18.64 ± 2.55^d^

Priming	Water	178.84 ± 3.34^ab^	7.80 ± 0.51^d^	13.94 ± 1.09	20.78 ± 3.22^cd^
	NaCl	183.41 ± 4.96^a^	8.25 ± 0.59^cd^	10.88 ± 1.01	16.43 ± 2.18^d^
	PEG	182.28 ± 4.13^a^	9.44 ± 0.37^abc^	12.35 ± 0.96	21.29 ± 4.32^cd^
	GA	169.43 ± 5.19^b^	8.70 ± 0.26^bcd^	11.14 ± 0.93	25.72 ± 4.56^bcd^

Presoaking	Water	178.03 ± 5.20^ab^	8.90 ± 0.40^ab^	13.57 ± 1.11	29.23 ± 4.38^abc^
	NaCl	178.87 ± 4.53^ab^	9.40 ± 0.37^ab^	13.02 ± 0.64	32.17 ± 4.26^ab^
	PEG	183.78 ± 3.68^a^	10.30 ± 0.36^a^	13.07 ± 0.79	37.13 ± 4.70^a^
	GA	182.16 ± 4.11^a^	9.55 ± 0.44^a^	13.58 ± 1.08	24.23 ± 2.57^bcd^

Different letters indicate significant differences from different pretreatments (*P* < 0.05).

## References

[1] Ritchie JT, Johnson A, Stewart BA, Nielsen DR (1990). Soil and plant factors affecting evaporation. *Irrigation of Agricultural Crops*.

[2] Budak H, Kantar M, Yucebilgili Kurtoglu K (2013). Drought tolerance in modern and wild wheat. *The Scientific World Journal*.

[3] Rosegrant MW, Agcaoili-Sombilla M, Perez ND (1995). *Food, Agriculture and the Environment Discussion Paper 5. Global Food Projections to 2020: Implications for Investment*.

[4] Heydecker W, Higgins J, Gulliver RL (1973). Accelerated germination by osmotic seed treatment. *Nature*.

[5] Passam HC, Kakouriotis D (1994). The effects of osmoconditioning on the germination, emergence and early plant growth of cucumber under saline conditions. *Scientia Horticulturae*.

[6] Sharifi RS, Khavazi K (2011). Effects of seed priming with Plant Growth Promoting Rhizobacteria (PGPR) on yield and yield attribute of maize (Zea mays L.) hybrids. *Journal of Food, Agriculture and Environment*.

[7] Bakht J, Shafi M, Jamal Y, Sher H (2011). Response of maize (*Zea mays* L.) to seed priming with NaCl and salinity stress. *Spanish Journal of Agricultural Research*.

[8] Chen K, Fessehaie A, Arora R (2012). Dehydrin metabolism is altered during seed osmopriming and subsequent germination under chilling and desiccation in *Spinacia oleracea* L. cv. Bloomsdale: possible role in stress tolerance. *Plant Science*.

[9] Dahal P, Bradford KJ (1990). Effects of priming and endosperm integrity on seed-germination rates of tomato genotypes. 2. Germination at reduced water potential. *Journal of Experimental Botany*.

[10] Finch-Savage WE, Dent KC, Clark LJ (2004). Soak conditions and temperature following sowing influence the response of maize (*Zea mays* L.) seeds to on-farm priming (pre-sowing seed soak). *Field Crops Research*.

[11] Adegbuyi E, Cooper SR, Don R (1981). Osmotic priming of some herbage grass seed using polyethylene glycol (PEG). *Seed Science and Technology*.

[12] Hurly RF, Van Staden J, Smith MT (1991). Improved germination in seeds of guayule (*Parthenium argentatum* Gray) following polyethylene glycol and gibberellic acid pretreatments. *Annals of Applied Biology*.

[13] Anosheh HP, Emam Y, Ashraf M (2014). Impact of cycocel on seed germination and growth in some commercial crops under osmotic stress conditions. *Archives of Agronomy and Soil Science*.

[14] Harris D, Pathan AK, Gothkar P, Joshi A, Chivasa W, Nyamudeza P (2001). On-farm seed priming: using participatory methods to revive and refine a key technology. *Agricultural Systems*.

[15] Sundstrom FJ, Reader RB, Edwards RL (1987). Effect of seed treatment and planting method on Tabasco pepper. *Journal of American Society for Horticultural Science*.

[16] Khan MA, Ungar IA (1984). The effect of salinity and temperature on the germination of polymorphic seeds and growth of *Atriplex triangularis* Willd. *American Journal of Botany*.

[17] Subedi KD, Ma BL (2005). Seed priming does not improve corn yield in a humid temperate environment. *Agronomy Journal*.

[18] Farahani HA, Moaveni P, Maroufi K (2011). Effect of hydropriming on germination percentage in sunflower (*Helianthus annus* L.) cultivars. *Advances in Environmental Biology*.

[19] Bradford KJ, Kigel J, Galili G (1995). Water relations in seed germination. *Seed Development and Germination*.

[20] Bradford KJ (1986). Manipulation of seed water relations via osmotic priming to improve germination under stress conditions. *HortScience*.

[21] Murungu FS, Chiduza C, Nyamugafata P, Clark LJ, Whalley WR, Finch-Savage WE (2004). Effects of on-farm seed priming on consecutive daily sowing occasions on the emergence and growth of maize in semi-arid Zimbabwe. *Field Crops Research*.

[22] Basra AS, Farooq M, Afzal I, Hussain M (2006). Influence of osmopriming on the germination and early seedling growth of coarse and fine rice. *International Journal of Agricultural Biology*.

[23] Hedden P, Phillips AL (2000). Gibberellin metabolism: new insights revealed by the genes. *Trends in Plant Science*.

[24] Sevik H, Guney K (2013). Effects of IAA, IBA, NAA, and GA_3_ on rooting and morphological features of *Melissa officinalis* L. stem cuttings. *The Scientific World Journal*.

[25] Zhang Y, Ni Z, Yao Y, Nie X, Sun Q (2007). Gibberellins and heterosis of plant height in wheat (*Triticum aestivum* L.). *BMC Genetics*.

[26] Gupta P, Mukherjee D (1982). Influence of GA3 pre-soaking of seeds on biochemical changes in seedling parts of *Pennisetum typhoides* Rich. *Proceedings of Indian National Science Academy B*.

[27] Guan B, Cao D, Yu JB (2014). Eco-physiological responses of seed germination of sweet sorghum to seed priming. *Chinese Journal of Ecology*.

[28] Farooq M, Aziz T, ur Rehman H, ur Rehman A, Cheema SA (2011). Evaluating surface drying and re-drying for wheat seed priming with polyamines: effects on emergence, early seedling growth and starch metabolism. *Acta Physiologiae Plantarum*.

[29] Farooq M, Basra SMA, Tabassum R, Afzal I (2006). Enhancing the performance of direct seeded fine rice by seed priming. *Plant Production Science*.

[30] Yang P, Li X, Wang X, Chen H, Chen F, Shen S (2007). Proteomic analysis of rice (*Oryza sativa*) seeds during germination. *Proteomics*.

[31] Chen K, Arora R (2013). Priming memory invokes seed stress-tolerance. *Environmental and Experimental Botany*.

[32] Harris D, Tripathi RS, Joshi A On-farm seed priming to improve crop establishment and yield in dry direct-seeded rice.

[33] Harris D, Tripathi RS, Joshi A, Pandey S, Mortimer M, Wade L, Tuong TP, Lopes K, Hardy B (2002). On-farm seed priming to improve crop establishment and yield in dry direct-seeded. *Direct Seeding: Research Strategies and Opportunities*.

